# circRNF13, a novel N^6^-methyladenosine-modified circular RNA, enhances radioresistance in cervical cancer by increasing CXCL1 mRNA stability

**DOI:** 10.1038/s41420-023-01557-0

**Published:** 2023-07-20

**Authors:** Junyu Shi, Xiaohui Rui, Chunxiao Han, Chaoping Wang, Lei Xu, Xiping Jiang

**Affiliations:** Department of Gynecology, The Third Affiliated Hospital of Soochow University, Changzhou, 213003 PR China

**Keywords:** Cervical cancer, Cervical cancer

## Abstract

**Background:**

Circular RNAs (circRNAs) and N^6^-methyladenosine (m^6^A) have been shown to play an increasingly critical role in the development of different cancers. However, there is limited evidence on how circRNAs and m^6^A interact to affect the radiosensitivity of cervical cancer (CC). This study provides a mechanistic understanding of the novel m^6^A-regulated circRNF13 in enhancing radioresistance in CC.

**Methods:**

Differentially expressed circRNAs were identified from radiosensitive and radioresistant CC tissues. Meanwhile, these circRNAs were subjected to methylated RNA immunoprecipitation (Me-RIP). Finally, the effects of these circRNAs on radiosensitivity were characterized.

**Results:**

CircRNF13 was poorly expressed in CC patients that were sensitive to concurrent radiochemotherapy. Experiments conducted both in vitro and in vivo confirmed that the knockdown of circRNF13 potentiated the radiosensitivity of CC cells. Further mechanistic studies revealed that METTL3/YTHDF2 promoted the degradation of circRNF13 and subsequently affected the stability of CXC motif chemokine ligand 1 (CXCL1), ultimately enhancing the radiosensitivity of CC cells.

**Conclusion:**

This study identified circRNF13 as a novel m^6^A-modified circRNA and validated the METTL3/YTHDF2/circRNF13/CXCL1 axis as a potential target for CC radiotherapy.

## Background

Globally, cervical cancer (CC) is the most common malignancy of the reproductive tract in females. According to the International Agency for Research on Cancer (IARC 2002) of the World Health Organization (WHO), it is estimated that 604,127 new cases and 341,832 annual deaths occurred worldwide in 2012, with 80–85% of cases occurring in developing countries [1]. Most patients with early-stage CC are treated with surgeries in the clinical scenario, while those with CC at the stage of IB2-IVA are recommended to receive synchronous chemoradiotherapy according to the National Comprehensive Cancer Network (NCCN) guidelines. Therefore, radiotherapy is a crucial component in the treatment of CC. However, for locally advanced CC, the five-year survival rates for stages IIB, IIIB, and IV are only 60–65%, 25–50%, and 20–35%, respectively. Moreover, approximately 40% of deaths are locally uncontrolled [2]. A retrospective study conducted by the European Society for Therapeutic Radiology and Oncology (ESTRO) has found that high-dose radiotherapy to high-risk clinical targets in CC could increase the 3-year local control rate to over 94%, 93%, and 86%, respectively. However, high-dose irradiation inevitably causes radiotoxicity to surrounding organs, such as the bladder, rectum, sigmoid colon, and vagina, and increases the risk of radiotherapy-associated complications [3]. As a result, current radiobiology and radiation oncology research is actively investigating ways to enhance the radiosensitivity of CC cells while minimizing the irradiation dose to normal peri-cervical tissues. This is an important area of study aimed at improving the effectiveness of treatment while reducing the side effects.

Circular RNAs (circRNAs) are a class of endogenous non-coding RNAs that are found in various eukaryotic cells [4]. The expression pattern and characteristics of circRNAs, including conservation, ubiquity, stability, and tissue/cell specificity, enable them to serve as biological markers in various types of tumors [5]. Many studies have demonstrated that circRNAs have the ability to serve as both biomarkers and therapeutic targets in CC, as they play important regulatory roles in the, migration, proliferation, and invasion of CC cells [6–9].

N^6^-methyladenosine (m^6^A), is an epitranscriptomic variation that occurs on the N6 position of adenosine. It is the most abundant modification in eukaryotic mRNA, accounting for approximately 0.1–0.4% of all adenosines [10]. Although discovered in the 1970s, it was not until recent years that the development of enzymology and high-throughput technology enabled researchers to establish a clear link between m^6^A dynamics and various diseases as well as developmental and cellular processes in eukaryotes. Of note, m^6^A is closely associated with cancer progression, including tumorigenesis, metastasis, and angiogenesis [11]. Evidence exists reporting that m^6^A modification levels and the associated m^6^A methyltransferases can regulate the radiosensitivity of tumors [11]. However, the specific mechanism and role of m^6^A-modified circRNAs in the radiosensitivity of CC are not well understood and require further investigation.

In this study, whole-genome sequencing was performed on three pre-treatment biopsies from radiotherapy-resistant CC patients (cervical lump regression less than 30% after synchronous chemoradiotherapy) and radiotherapy-sensitive CC patients (cervical lump regression to complete clinical response (CR) after synchronous chemoradiotherapy). Based on the selected differentially expressed circRNAs, methylated RNA immunoprecipitation (Me-RIP) and radiosensitivity validation were subsequently performed, which identified that circRNF13 increased the radioresistance of CC cells. Furthermore, METTL3/YTHDF2 promoted the degradation of circRNF13, which in turn affected the stability of CXC motif chemokine ligand 1 (CXCL1) and ultimately enhanced the radiosensitivity of CC cells.

## Results

### Screening and characterization of circRNF13 in CC

**Fig. 1:**
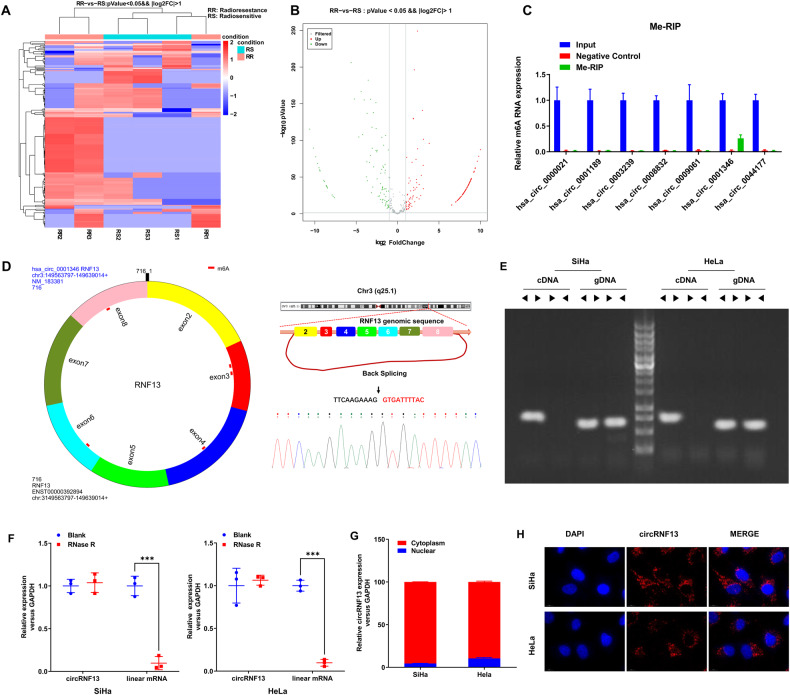
Screening and characteristics of m6A-related circRNF13 in cervical cancer. **A**, **B** After the gene sequencing of the radiosensitive and radioresistant tissues of cervical cancer, the heat map and volcano plot (Fold Change > 2, *P* < 0.05) of the differential circRNAs were analyzed. **C** Me-RIP screened the methylation modification of candidate circRNAs. **D** hsa_circ_0001346 is formed by the circularization of RNF13 gene, which was verified by Sanger sequencing. **E** cDNA and genomic gDNA reverse transcribed from circRNF13 were subjected to PCR amplification in SiHa and HeLa cells. **F** Differences between circRNF13 and linear RNA upon RNase R treatment in SiHa and HeLa cells. **G** Nucleocytoplasmic separation assay to characterize the distribution of circRNF13 in SiHa and HeLa cells. **H** The distribution of circRNF13 was examined by FISH assay in SiHa and HeLa. (***P < 0.001, **P < 0.01, *P < 0.05).

In order to evaluate the effects of m^6^A-modified circRNAs on the radiosensitivity of CC, the whole-genome sequencing was performed on six biopsy specimens of CC prior to radical surgery and synchronous chemoradiotherapy. These samples were histopathologically confirmed with squamous carcinoma of the cervix, and three cases with cervical lump regression to clinical CR after synchronous chemoradiotherapy were used as the radiosensitive group, and three cases with cervical lump regression less than 30% after synchronous chemoradiotherapy were used as the radioresistant group. A total of 2664 new circRNAs were detected, with an overall length of 6944600 nt and an average length of 2606.83 nt. From these, 247 circRNAs exhibiting significant differences between radioresistant and radiosensitive tissues were selected (Fold Change > 2, *P* < 0.05) (Fig. 1A-B). CircRNAs that were expressed in only one or two specimens were removed due to detection errors. In addition, the circRNAs with a length greater than 5000 bp were also removed. Ultimately, seven differentially expressed circRNAs were selected for further analysis, of which five were highly expressed in radiosensitive CC tissues (hsa_circ_0000021, hsa_circ_0001189, hsa_circ_0003239, hsa_circ_0008832, and hsa_circ_0009061) and two were highly expressed in radioresistant CC tissues (hsa_circ_0001346 and hsa_circ_0044177). Furthermore, it was investigated if these seven circRNAs had m^6^A methylation modifications by examining the presence of such modifications in CC cells (HeLa) *via* the Me-RIP assay. It was found that m^6^A methylation modification occurred in hsa_circ_0001346 (Fig. 1C). Therefore, hsa_circ_0001346 was selected for subsequent studies.

The circBase database analysis demonstrated that hsa_circ_0001346, with a spliced length of 716 nt, was located at chr3:149563797-149639014. It was produced by the cyclization of exons 2–8 of the *RNF13* gene. It was predicted that circRNF13 might have five sites of m^6^A methylation (sites: 74, 190, 276, and 667), hereafter referred to as circRNF13, which was further validated by Sanger sequencing (Fig. 1D). Convergent and divergent primers were used to substantiate the circular structure of circRNF13 (Fig. 1E). After RNase R treatment of total RNA from HeLa and SiHa cells, circRNF13 was found to be more stable than linear RNA (Fig. 1F). The nuclear-cytoplasmic fractionation and FISH assays demonstrated that circRNF13 was predominantly present in the cytoplasm of CC cells (Fig. 1G, H). Collectively, these experimental results indicated that circRNF13 had a circular structure and exhibited stable expression in the cytoplasm of CC cells.

### Inhibition of circRNF13 increases radiosensitivity of CC in vitro and in vivo

**Fig. 2:**
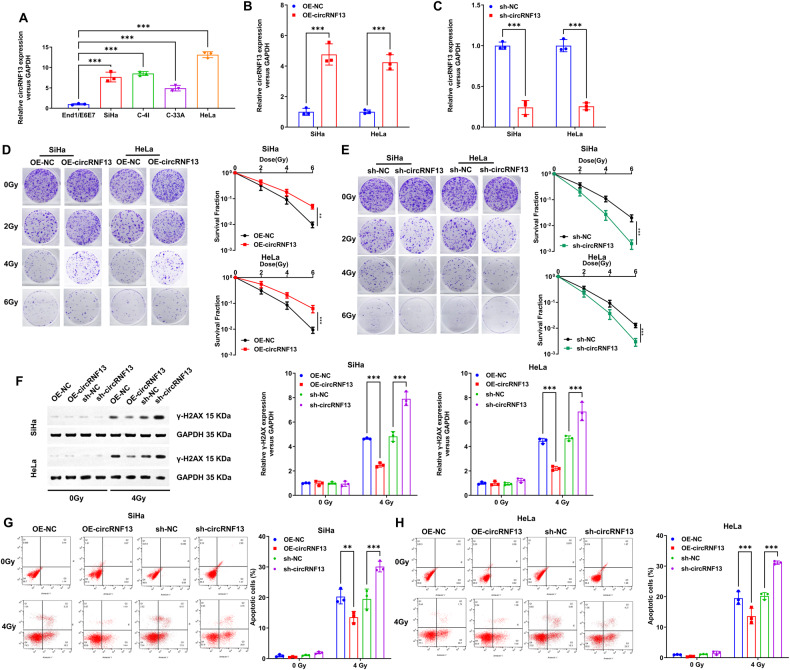
CircRNF13 enhances the radioresistance of cervical cancer cells in vitro. **A** qRT-PCR to detect circRNF13 expression in End1/E6E7, SiHa, C-4I, HeLa and C-33A cells. **B**, **C** qRT-PCR to detect circRNF13 expression in SiHa and HeLa with ectopic expression or silencing of circRNF13. **D** Colony formation assay to detect the colony formation number of cells after 0, 2, 4 and 6 Gy irradiation in SiHa and HeLa cells transfected with OE-NC and OE-circRNF13. **E** Colony formation assay to detect the colony formation number of cells after 0, 2, 4 and 6 Gy irradiation in SiHa and HeLa cells transfected with sh-NC and sh-circRNF13. **F** Western blot detected γ-H2AX expression in circRNF13-overexpressed or circRNF13-depleted SiHa and HeLa cells after 4 Gy irradiation. **G**, **H** After 4 Gy irradiation, apoptosis of circRNF13-overexpressed or circRNF13-depleted SiHa and HeLa cells was examined by flow cytometry. (***P < 0.001, **P < 0.01, *P < 0.05).

In the initial experimental investigations, the expression of circRNF13 was examined in both normal cervical epithelial cells (End1/E6E7) and various CC cell lines (SiHa, C-4I, HeLa, and C-33A). The outcomes of this experiment demonstrated that the expression level of circRNF13 was higher in the CC cell lines as compared to the normal cervical epithelial cells (Fig. 2A). Since human papillomavirus (HPV) type 16 and 18 infections are the main cause of CC, HPV-16-positive SiHa cells and HPV-18-positive HeLa cells were selected for the following cellular experiments.

The CC cell lines were constructed with ectopic expression or silencing of circRNF13. The levels of circRNF13 expression in SiHa and HeLa cells were examined following transfection (Fig. 2B, C). Subsequent colony formation assay revealed that reducing circRNF13 expression resulted in a significant decrease in cell survival after irradiation while increasing its expression had the opposite effect, that is, elevated cell survival (Fig. 2D, E). Similarly, Western blotting analysis showed that the expression of DNA damage-related protein γ-H2AX was considerably higher in cells with down-regulated circRNF13, while it was significantly decreased in cells overexpressing circRNF13 (Fig. 2F). In addition, the AnnexinV/PI-based apoptosis assay also suggested that apoptosis was significantly elevated in cells with down-regulated circRNF13, whereas it was significantly decreased in cells overexpressing circRNF13 (Fig. 2G, H).

**Fig. 3:**
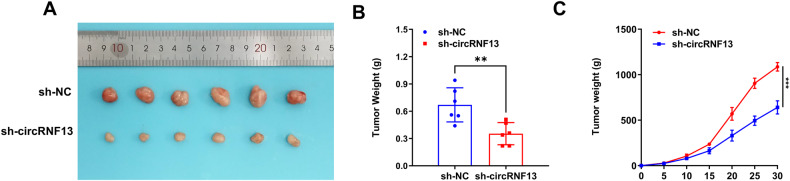
CircRNF13 enhances the radioresistance of cervical cancer cells in vivo. **A** Representative images of collected tumors. **B** Subcutaneous tumor weights in tumor-bearing mice. **C** Growth curve of subcutaneous tumor in tumor-bearing mice. (***P < 0.001, **P < 0.01, *P < 0.05).

For further evaluation of the effect of circRNF13 on the radiosensitivity of CC in vivo, with a control group and a circRNF13 down-regulation group in SiHa, a subcutaneous xenograft model in nude mice was established. When the tumor of nude mice reached 200 mm^3^, a single dose of 15 Gy of irradiation was given. Subsequently, the volume of the tumors was measured every five days to track their growth over time. The nude mice were anesthetized and sacrificed after 30 days of irradiation. The tumors of the mice were removed (Fig. 3A). The tumor weights of mice in the presence of circRNF13 down-regulation were remarkably reduced relative to those of control mice (Fig. 3B). In addition, curbed tumor growth rates and reduced volumes were observed in nude mice in response to circRNF13 down-regulation (Fig. 3C). According to these outcomes, the inhibition of circRNF13 enhanced the radiosensitivity of CC in both in vivo and in vitro experiments.

### Expression of CircRNF13 is regulated by the METTL3 /YTHDF2-mediated m^6^A methylation

**Fig. 4:**
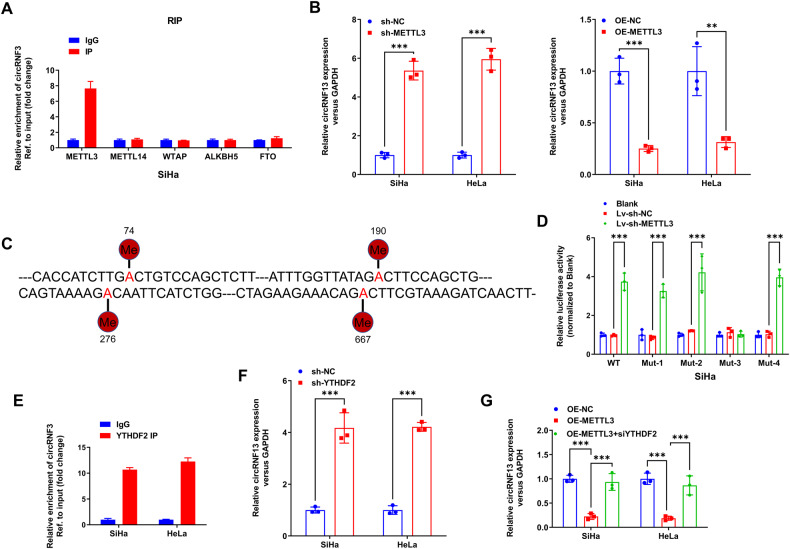
Expression of CircRNF13 is regulated by the METTL3 /YTHDF2-mediated m6A methylation. **A** RIP assay was used to detect which m^6^A enzymes were involved in the regulation of circRNF13 in SiHa. **B** qRT-PCR to detect circRNF13 expression in SiHa and HeLa with ectopic expression or silencing of METTL3. **C** The constructed luciferase reporter plasmids by inserting partial wildtype circRNF13 sequences or mutated m6A sites (M1-M4) circRNF13 sequences. **D** The luciferase reporters with wildtype or mutant plasmids were transfected into SiHa cells (with the down-regulated METTL3), followed by the measurement of luciferase activity. **E** RIP assay to detect the combination between YTHDF2 and circRNF13. **F** qRT-PCR to detect circRNF13 expression in SiHa and HeLa transfected down-regulated YTHDF2. **G** qRT-PCR to detect circRNF13 expression in SiHa and HeLa transfected up-regulated METTL3 or/both down-regulated YTHDF2. (***P < 0.001, **P < 0.01, *P < 0.05).

The dynamic regulation of m^6^A modifications is primarily attributed to the activity of demethylases such as ALKBH5 and FTO and methyltransferases, including, METTL3, METTL14, and WTAP. Since the effects of m^6^A enzymes are commonly achieved by binding to the corresponding transcripts, RIP assay was used to detect which m^6^A enzymes were involved in the regulation of circRNF13 in SiHa. It was found that METTL3 could enrich the RNA fragments of circRNF13, while METTL14, WTAP, ALKBH5, and FTO did not exhibit a significant binding affinity for RNA fragments in circRNF13 (Fig. 4A). Therefore, METTL3 might be the major m^6^A modifying enzyme of circRNF13. Further studies discovered that the expression of circRNF13 was significantly diminished in the METTL3-overexpressing CC cells, whereas opposite results were witnessed in CC cells with down-regulated METTL3 (Fig. 4B).

The focus of the study then shifted towards confirming the essential function of m^6^A methylation in the regulation of circRNF13 by METTL3 as well as identifying the specific m^6^A methylation sites of circRNF13. SRAMP software (http://www.cuilab.cn/sramp/) was used for the sequence prediction of its high-confidence m^6^A sites. Subsequently, luciferase reporter plasmids were constructed by introducing partial sequences of circRNF13 that contained either wild-type or mutant m6A sites (MUT1: mutant site 74; MUT2: mutant site 190; MUT3: mutant site 276; MUT4: mutant site 667) (Fig. 4C). When METTL3 was down-regulated, the activity of luciferase was reduced in SiHa cells which were transfected with wild-type plasmids and mutant plasmids (MUT1, MUT2, MUT3, and MUT4). However, the luciferase activity of circRNF13 containing MUT3 remained unaffected (Fig. 4D). It was confirmed that the regulation of circRNF13 by METTL3 was dependent on m^6^A methylation and that the principal m^6^A methylation site of circRNF13 was 276.

The main m^6^A-associated reader proteins, including, YTHDC1/2, IGF2BP1/2/3, and YTHDF1/2/3. The prior data indicated that m^6^A modification down-regulated circRNF13 expression, hence leading to the speculation that m^6^A modification may affect circRNA stability. It is interesting to note that YTHDF2 has been widely documented to be involved in regulating the stability of m^6^A-containing mRNA [12, 13]. Recent studies have also highlighted its ability to initiate the decay of m^6^A-containing circRNAs [14, 15]. Subsequently, it was confirmed in the RIP assay that YTHDF2 could bind to circRNF13 (Fig. 4E)., When YTHDF2 was knocked down in SiHa and HeLa cells, the expression of circRNF13 was up-regulated (Fig. 4F). In addition, YTHDF2 knockdown counteracted the down-regulation of circRNF13 caused by METTL3 overexpression (Fig. 4G). Therefore, circRNF13 expression was confirmed to be regulated by METTL3/YTHDF2-mediated m^6^A methylation.

### CircRNF13 promotes radioresistance in CC by increasing the stability of CXCL1 mRNA

**Fig. 5:**
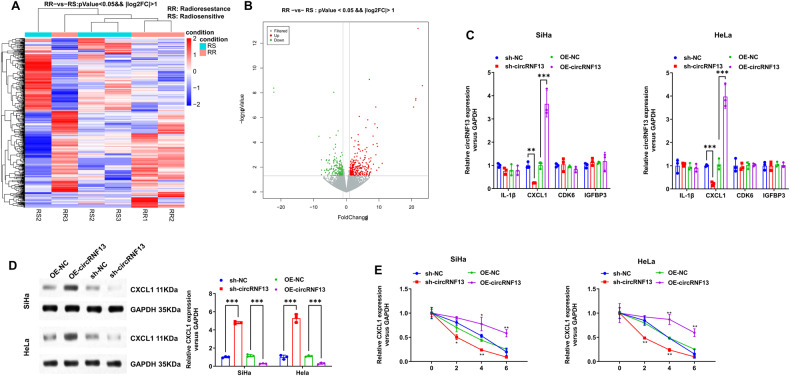
CircRNF13 increase the stability of CXCL1 mRNA. **A**, **B** After the gene sequencing of the radiosensitive and radioresistant tissues of cervical cancer, the heat map and volcano plot (Fold Change > 2, *P* < 0.05) of the differential mRNAs were analyzed. **C** qRT-PCR to detect IL1B, CXCL1, CDK6, and IGFBP3 expression in SiHa and HeLa with ectopic expression or silencing of circRNF13. **D** Western blot detected CXCL1 expression in circRNF13-overexpressed or circRNF13-depleted SiHa and HeLa cells. **E** circRNF13-overexpressed or circRNF13-depleted cells were treated with actinomycin D (10 μg/mL) and collected at the point of 0, 2, 4 and 6 h, followed by the calculation of RNA decay rate. (***P < 0.001, **P < 0.01, *P < 0.05).

**Fig. 6:**
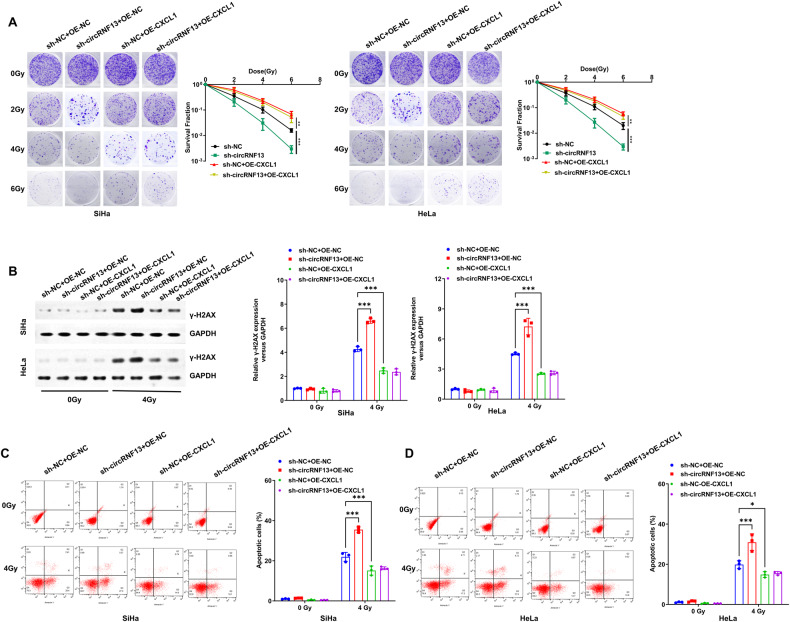
CircRNF13 promotes radioresistance in CC by increasing the stability of CXCL1 mRNA. **A** Colony formation assay to detect the colony formation number of cells after 0, 2, 4 and 6 Gy irradiation in SiHa and HeLa cells transfected with sh-NC + OE-NC, sh-circRNF13+OE-NC, sh-NC + OE-CXCL1 and sh-circRNF13+OE-CXCL1. **B** Western blot detected γ-H2AX expression in SiHa and HeLa cells transfected with sh-NC + OE-NC, sh-circRNF13+OE-NC, sh-NC + OE-CXCL1 and sh-circRNF13+OE-CXCL1 after 4 Gy irradiation. **C**, **D** Flow cytometry to detect the apoptosis of SiHa and HeLa cells transfected with sh-NC + OE-NC, sh-circRNF13+OE-NC, sh-NC + OE-CXCL1 and sh-circRNF13+OE-CXCL1 after 4 Gy irradiation. (***P < 0.001, **P < 0.01, *P < 0.05).

Finally, the above-mentioned batch of specimens was subjected to high-throughput sequencing for mRNAs to further investigate the downstream regulatory genes or signaling pathways of circRNF13. 490 mRNAs with significant differences in radioresistant and radiosensitive tissues were then selected (Fold Change > 2, *P* < 0.05) (Fig. 5A, B). Correlation analyses of circRNF13 with these differentially expressed genes were performed, and 77 mRNAs showed a strong association with circRNF13 (r > 0.90, *P* < 0.05). Based on previously reported studies, it has been documented that IL1B, CXCL1, CDK6, and IGFBP3 were closely associated with tumor radiosensitivity. Either down-regulation or overexpression of circRNF13 in CC cell lines was performed. Among these mRNAs, only the expression of CXCL1 mRNA and protein was significantly altered (Fig. 5C, D). Down-regulation of circRNF13 expression decreased the stability of CXCL1 mRNA, while circRNF13 overexpression restored the stability of CXCL1 mRNA (Fig. 5E). In vitro rescue cloning experiments revealed that the increased cell death caused by downregulation of circRNF13 can be reversed by upregulating CXCL1 (Fig. 6A). Similarly, the enhanced expression of γ-H2AX caused by downregulation of circRNF13 can also be reversed by upregulating CXCL1 (Fig. 6B). The increased cell apoptosis caused by downregulation of circRNF13 can also be reversed by upregulating CXCL1 (Fig. 6C, D). Collectively, these findings substantiated that circRNF13 augmented radioresistance in CC by increasing the stability of CXCL1 mRNA.

## Discussion

Recent studies have shown such novel regulatory mechanistic understanding between circRNAs and m^6^A as circRNA-m^6^A interactions [16]. m^6^A methylation is one of the most common forms of modification in RNA molecules. It plays a vital role in many physiological processes, including transcriptional and post-transcriptional regulation, RNA shearing, RNA stability, translation, and RNA localization [10]. It is worth noting that circRNAs are implicated in numerous biological processes related to carcinogenesis and have important functions in the radiosensitivity of tumors. CircRNAs interact with miRNAs or RNA-binding proteins (RBPs) as a "sponge" structure, which mediates mRNA stability, transcription rate, and post-translational modifications, ultimately affecting tumor radiosensitivity [17]. In the regulatory mechanism of circRNA-m^6^A, m^6^A modification can affect the production, processing, and stability of circRNAs, which further regulates the function of circRNAs in cells. For example, m^6^A can promote the shearing and degradation of circRNAs [18]. In addition, m^6^A can also recruit RBPs to bind to circRNAs, further regulating its role in cells [19]. In contrast, circRNAs can also regulate the modification pattern of m^6^A by interacting with each other. For example, some studies have suggested that circRNAs can regulate the level of m^6^A modification by mediating m^6^A methyltransferases, thereby affecting RNA metabolism and functions [20, 21]. The present study demonstrated that circRNF13 expression was regulated by m^6^A methylation of METTL3/YTHDF2 and that circRNF13 mediated radioresistance in CC through increasing CXCL1 mRNA stability.

In recent years, multiple studies have demonstrated that circRNF13 has some important biological functions in a variety of tumors, and these functions may vary depending on the type of tumors [22–26]. For example, lung cancer and nasopharyngeal carcinoma showed a remarkably reduced expression level of circRNF13 and also showed a close association with the clinical characteristics of these individuals (e.g., tumor size, tumor-node-metastasis [TNM] stage, and lymph node metastasis) [22, 25]. Interestingly, circRNF13 prolongs the half-life of SUMO2 mRNA by binding to the *SUMO2* gene, which in turn inhibits the proliferation and metastatic ability of nasopharyngeal carcinoma [22]. However, overexpression of circRNF13 was observed in pancreatic cancer, involved in promoting its malignant ability [23, 24]. Further mechanistic studies revealed that circRNF13 achieves this by acting as a miR-654-3p sponge to promote PDK3 level [23]. In this research, it was discovered that circRNF13 expression was high in radioresistant CC tissues. Additionally, it was found that inhibiting circRNF13 increased in vitro and in vivo radiosensitivity of CC.

The interaction between m^6^A modifications and circRNAs is a promising subject. The METTL3/YTHDC1-mediated m^6^A modification is crucial in the formation of circRNAs with encoding protein potential [27, 28]. Additionally, m^6^A was shown to assist in the translation of endogenous circRNAs with the help of eIF4G2 and YTHDF3 [29]. Furthermore, nucleoplasmic translocation of m^6^A-modified circRNAs might be achieved through a YTHDC1-dependent manner [18]. Meanwhile, YTHDF2 has been shown to promote the degradation of circRNAs [15, 30]. Results of the present study suggested that METTL3/YTHDF2 plays a crucial role in regulating the stability of circRNF13.

CXCL1 is a small molecule cytokine belonging to the family of CXC chemokines, located on human chromosome 4 [31]. Previous evidence has indicated that CXCL1 plays a role in the growth, tumorigenesis, and metastasis of various tumors. CXCL1 is highly expressed in CC, and high CXCL1 expression corresponds to an unfavorable prognosis in patients with CC [32–34]. Down-regulation of CXCL1 expression could result in decreased proliferation and migration of CC cells and an increase in apoptosis [32]. Intriguingly, multiple studies have demonstrated that CXCL1 is closely associated with tumor radiosensitivity [35, 36]. Tumor-associated fibroblasts, an important component of the tumor environment, have been found to cause malignant transformation of normal fibroblasts and inhibit reactive oxygen species through the secretion of CXCL1. This CXCL1 secretion leads to a decrease in enzyme activity, increased DNA damage repair, and, ultimately, tumor radioresistance [36]. In the present study, it was confirmed that CXCL1 was highly expressed in radioresistant CC tissues. Moreover, it has been found that CXCL1 can reverse the radiosensitivity induced by circRNF13 inhibition.

**Fig. 7:**
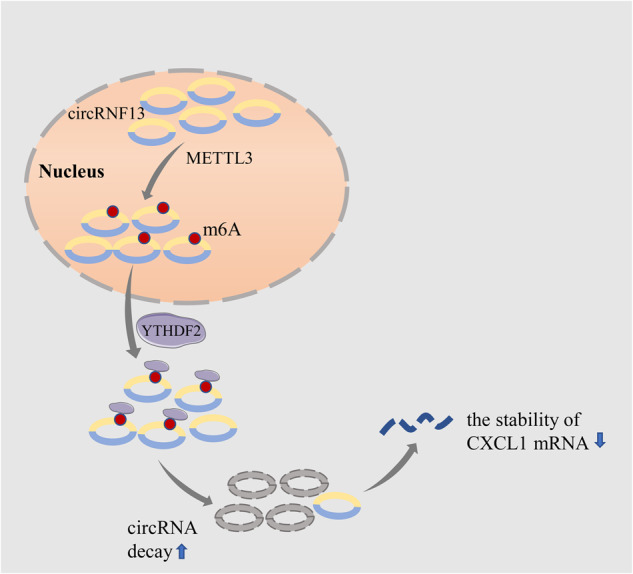
Schematic diagram depicts the proposed mechanisms of circRNF13 in cervical cancer.

In conclusion, this work demonstrated that circRNF13, a novel m^6^A-modified circular RNA, enhanced CC radioresistance by increasing CXCL1 mRNA stability (Fig. 7). The obtained results provided novel insights into the regulatory mechanism of the METTL3/YTHDF2/circRNF13/CXCL1 axis, which affects the radiosensitivity of CC cells. Moreover, this study offers a feasibility and theoretical basis for utilizing circRNF13 as a diagnostic and therapeutic target for screening suitable CC individuals and improving radiosensitivity during synchronous chemoradiotherapy. This approach could offer a new avenue for managing CC patients and enhancing their treatment outcomes.

## Materials and methods

### Tissue sample collection

The tissue samples for this study were collected from individuals with CC prior to receiving synchronous chemoradiotherapy for CC from the Third Affiliated Hospital of Soochow University. None of the enrolled individuals with CC had received any anti-tumor therapies before sample collection. To evaluate the therapeutic effect of synchronous chemoradiotherapy on CC, imaging examinations such as computed tomography (CT) and magnetic resonance imaging (MRI) were used. Two pathologists from the Third Affiliated Hospital of Soochow University provided the pathological diagnostic reports for the specimens. Prior to specimen collection, individuals with CC and their families were informed during pre-treatment conversations, and they signed a consent form. The study procedure was approved by the Ethics Committee of the Third Affiliated Hospital of Soochow University and compiled with the *Declaration of Helsinki*. (Approval No. [2021] Ethical Review Institute No. 103).

### Cell culture

The CC cell lines (SiHa, C-4I, HeLa, C-33A) and the normal cervical epithelial cell line End1/E6E7 were obtained from the American Type Culture Collection (ATCC, Manassas, VA, USA). These cell lines were cultured in DMEM supplemented with 1% streptomycin-antibiotic mixture and 10% fetal bovine serum (FBS), and incubated at 37 °C with 5% CO_2_. Cells were regularly subcultured every 2–3 days until they reached the logarithmic growth phase.

### Data accessibility

The RNA-seq data that underlie the findings of this study have been archived in the National Genomics Data Center (https://ngdc.cncb.ac.cn/search/?dbId=hra&q = HRA004321&page=1). The accession number is HRA004321.

### Quantitative real-time PCR analysis (qRT-PCR)

RNA extraction from cells and tissues was performed using TRIzol reagent, and the concentration and quality of the extracted RNA were assessed using a NanoDrop2000 microspectrophotometer. Subsequently, cDNA was synthesized from the RNA using a PrimeScript RT Reagent Kit through reverse transcription. Real-time fluorescence quantitative PCR was then conducted using TB Green Fast qPCR Mix to quantify the expression of circRNA and mRNA. The housekeeping gene glyceraldehyde-3-phosphate dehydrogenase (GAPDH) was used as the internal reference for both circRNA and mRNA. The expression levels of circRNA and mRNA were analyzed using the 2^-ΔΔCt^ method. The primer sequences used for PCR are listed in Supplementary Table 1.

### RNA immunoprecipitation (RIP) assay

The RIP assay was conducted using the Magna RIP Kit (Millipore, Bedford, MA, USA). Magnetic beads were pre-coated with 5 mg antibodies specific to METTL3, METTL14, YTHDF2, WTAP, ALKBH5, FTO, YTHDF2, and corresponding immunoglobulin G (IgG), following the provided guidelines. These coated beads were then incubated with cell lysates and treated with proteinase K to degrade the proteins. Once the complex formed, it was collected by precipitation and separated from the beads. The extracted RNAs were purified for subsequent qPCR analysis. The level of enrichment was determined by normalizing the input.

### Methylated RIP (Me-RIP) qPCR

Me-RIP was conducted using the RIP kit (17-10499, Merck Millipore) following the manufacturer’s protocols. Cell lysis was carried out using 1 mL of RIP lysis buffer. After a 10 min incubation, 100 μL of the lysate was extracted and stored at −80 °C. Subsequently, the anti-m6A antibody (diluted at 1:50) or normal rabbit IgG (diluted at 1:50) was mixed with protein A/G microbeads and incubated for 2 h at 4 °C. Finally, RNA analysis was performed using qRT-PCR, following the same procedures as mentioned above.

### RNase R Resistance assay

Total RNA (2 μg) was isolated and incubated at 37 °C for 1 h in the presence or absence of RNase R (5 U/μg, R7092L, Beyotime, Shanghai, China). The level of circRNF13 remaining after the treatment was determined using qRT-PCR.

### Nuclear-cytoplasmic fractionation

Total RNA was extracted from the cells using TRIzol reagent (Takara, Dalian, China). To fractionate the nucleus and cytoplasm of SiHa and HeLa cells, Cytoplasmic and Nuclear Extraction Reagents (Thermo Fisher Scientific, Waltham, MA, USA) were employed. The cytoplasmic fraction was labeled using GAPDH, while the nuclear fraction was labeled using U6.

### Western blotting

Western blotting analysis was performed following previously established methods. The antibodies used in this study were: anti-γ-H2AX antibody (1:2000; Ab11174, Abcam, Cambridge, MA, USA), anti-CXCL1 antibody (1:500, ab206411, Abcam), and anti-GAPDH (1:8000, AC035, ABclonal, China). The Pierce™ ECL Western Blotting Substrate Kit (Thermo Fisher Scientific) was utilized to visualize the bands. Subsequently, the blots were exposed using the Tanon 4600 Automatic Chemiluminescence Imaging Analysis System (Tanon, Shanghai, China).

### Colony formation assay

Transfected cells were collected and seeded into 6-well plates. After plating, the cells were exposed to different doses of 6MV X-ray radiation. The cells were then cultured for 10–14 days. Following the incubation period, colony fixation was carried out using methanol, and the colonies were counted after staining with 0.5% crystal violet (dissolved in 20% methanol).

### Apoptosis detection

Apoptotic cell death was assessed using the Annexin V/PI apoptosis kit (Beyotime). Approximately 1 × 10 ^ 5 cells were stained with Annexin V/PI and incubated for 48 h. Subsequently, the cells were analyzed using two-color flow cytometry under low-light conditions. Early apoptotic cells were detected by green fluorescence, while late apoptotic cells exhibited both red and green fluorescence. Non-apoptotic, viable cells displayed minimal or no fluorescence after treatment with the Annexin V/PI probes.

### Luciferase report assay

In order to assess the impact of m6A modification on circRNF13, six plasmids containing partial circRNF13 sequences were generated. These plasmids were designed to harbor either mutant or wild-type m6A sites (M1-M5). For the luciferase reporter assays, SiHa cells overexpressing METTL3 were cultured in 24-well plates at a density of 1 × 10 ^ 5 cells/well. The plasmids were transfected into the cells using Lipofectamine 3000 (Thermo Fisher Scientific, USA). After 24 h, the luciferase activity was evaluated using the Dual-Luciferase Reporter Assay System (Promega, San Luis Obispo, CA, USA) and normalized to the renilla luciferase activity.

### Animal experiment

Animal experiments were conducted following the guidelines and procedures established by the Animal Management and Use Committee of Soochow University, and the study was approved by the Ethics Committee of the Third Affiliated Hospital of Soochow University. Due to funding and equipment limitations, this experiment uses a group of 6 mice for each set. Experimental animals were randomly divided into a control group and a circRNF13 down-regulation group. No blinding was done. Stably transfected cell lines were created by silencing circRNF13 in CC SiHa cells. Once xenografts were established, the tumors reached an approximate volume of 200 mm^3^. A single dose of 15 Gy irradiation was administered to female BALB/c nude mice (4–5 weeks old) in the murine model. The tumor volume was measured and recorded using vernier calipers every five days after irradiation. After 30 days, the mice were euthanized under anesthesia, and tumor tissue was collected for further investigations.

### Statistical analysis

Statistical analysis was performed using SPSS 21.0 software (IBM, Armonk, NY, USA). The measurement results were presented as mean ± standard deviation (mean ± SD). The t-test was used to compare continuous variables between two groups, while analysis of variance (ANOVA) was employed to compare continuous variables among multiple groups. The difference was shown to be statistically significant when *P*-value was lower than 0.05.

## Supplementary information

Supplementary table 1

Supplemental Material- Original Blots

## Data Availability

The RNA-seq data that underlie the findings of this study have been archived in the National Genomics Data Center (https://ngdc.cncb.ac.cn/search/?dbId=hra&q = HRA004321&page=1). The accession number is HRA004321.

## References

[1] Sung H, Ferlay J, Siegel RL, Laversanne M, Soerjomataram I, Jemal A (2021). Global Cancer Statistics 2020: GLOBOCAN Estimates of Incidence and Mortality Worldwide for 36 Cancers in 185 Countries. CA Cancer J Clin.

[2] Tsu V, Jeronimo J (2016). Saving the World’s Women from Cervical Cancer. N Engl J Med.

[3] Tanderup K, Fokdal LU, Sturdza A, Haie-Meder C, Mazeron R, van Limbergen E (2016). Effect of tumor dose, volume and overall treatment time on local control after radiochemotherapy including MRI guided brachytherapy of locally advanced cervical cancer. Radiother Oncol.

[4] Memczak S, Jens M, Elefsinioti A, Torti F, Krueger J, Rybak A (2013). Circular RNAs are a large class of animal RNAs with regulatory potency. Nature.

[5] Rybak-Wolf A, Stottmeister C, Glazar P, Jens M, Pino N, Giusti S (2015). Circular RNAs in the mammalian brain are highly abundant, conserved, and dynamically expressed. Mol Cell.

[6] Zhang C, Liu P, Huang J, Liao Y, Pan C, Liu J (2021). Circular RNA hsa_circ_0043280 inhibits cervical cancer tumor growth and metastasis via miR-203a-3p/PAQR3 axis. Cell Death Dis.

[7] Li X, Ma N, Zhang Y, Wei H, Zhang H, Pang X (2020). Circular RNA circNRIP1 promotes migration and invasion in cervical cancer by sponging miR-629-3p and regulating the PTP4A1/ERK1/2 pathway. Cell Death Dis.

[8] Rong X, Gao W, Yang X, Guo J (2019). Downregulation of hsa_circ_0007534 restricts the proliferation and invasion of cervical cancer through regulating miR-498/BMI-1 signaling. Life Sci.

[9] Chen Y, Geng Y, Huang J, Xi D, Xu G, Gu W (2021). CircNEIL3 promotes cervical cancer cell proliferation by adsorbing miR-137 and upregulating KLF12. Cancer Cell Int.

[10] Fu Y, Dominissini D, Rechavi G, He C (2014). Gene expression regulation mediated through reversible m(6)A RNA methylation. Nat Rev Genet.

[11] Huang J, Shao Y, Gu W (2021). Function and clinical significance of N6-methyladenosine in digestive system tumours. Exp Hematol Oncol.

[12] Wang X, Lu Z, Gomez A, Hon GC, Yue Y, Han D (2014). N6-methyladenosine-dependent regulation of messenger RNA stability. Nature.

[13] Lee Y, Choe J, Park OH, Kim YK (2020). Molecular Mechanisms Driving mRNA Degradation by m(6)A Modification. Trends Genet.

[14] Zhou C, Molinie B, Daneshvar K, Pondick JV, Wang J, Van Wittenberghe N (2017). Genome-Wide Maps of m6A circRNAs Identify Widespread and Cell-Type-Specific Methylation Patterns that Are Distinct from mRNAs. Cell Rep.

[15] Park OH, Ha H, Lee Y, Boo SH, Kwon DH, Song HK (2019). Endoribonucleolytic Cleavage of m(6)A-Containing RNAs by RNase P/MRP Complex. Mol Cell.

[16] Huang H, Weng H, Chen J (2020). m(6)A Modification in Coding and Non-coding RNAs: Roles and Therapeutic Implications in Cancer. Cancer Cell.

[17] Huang J, Sun H, Chen Z, Shao Y, Gu W (2022). Mechanism and Function of Circular RNA in Regulating Solid Tumor Radiosensitivity. Int J Mol Sci.

[18] Chen RX, Chen X, Xia LP, Zhang JX, Pan ZZ, Ma XD (2019). N(6)-methyladenosine modification of circNSUN2 facilitates cytoplasmic export and stabilizes HMGA2 to promote colorectal liver metastasis. Nat Commun.

[19] Lin H, Wang Y, Wang P, Long F, Wang T (2022). Mutual regulation between N6-methyladenosine (m6A) modification and circular RNAs in cancer: impacts on therapeutic resistance. Mol Cancer.

[20] Liu L, Gu M, Ma J, Wang Y, Li M, Wang H (2022). CircGPR137B/miR-4739/FTO feedback loop suppresses tumorigenesis and metastasis of hepatocellular carcinoma. Mol Cancer.

[21] Yao B, Zhang Q, Yang Z, An F, Nie H, Wang H (2022). CircEZH2/miR-133b/IGF2BP2 aggravates colorectal cancer progression via enhancing the stability of m(6)A-modified CREB1 mRNA. Mol Cancer.

[22] Mo Y, Wang Y, Zhang S, Xiong F, Yan Q, Jiang X (2021). Circular RNA circRNF13 inhibits proliferation and metastasis of nasopharyngeal carcinoma via SUMO2. Mol Cancer.

[23] Zhao Q, Zhu Z, Xiao W, Zong G, Wang C, Jiang W (2022). Hypoxia-induced circRNF13 promotes the progression and glycolysis of pancreatic cancer. Exp Mol Med.

[24] Liu X, Zhou L, Chen Y, Jiang X, Jiang J (2021). CircRNF13 Promotes the Malignant Progression of Pancreatic Cancer through Targeting miR-139-5p/IGF1R Axis. J Oncol.

[25] Wang L, Liu S, Mao Y, Xu J, Yang S, Shen H (2018). CircRNF13 regulates the invasion and metastasis in lung adenocarcinoma by targeting miR-93-5p. Gene.

[26] Chen Y, Li S, Wei Y, Xu Z, Wu X (2021). Circ-RNF13, as an oncogene, regulates malignant progression of HBV-associated hepatocellular carcinoma cells and HBV infection through ceRNA pathway of circ-RNF13/miR-424-5p/TGIF2. Bosn J Basic Med Sci.

[27] Tang C, Xie Y, Yu T, Liu N, Wang Z, Woolsey RJ (2020). m(6)A-dependent biogenesis of circular RNAs in male germ cells. Cell Res.

[28] Di Timoteo G, Dattilo D, Centron-Broco A, Colantoni A, Guarnacci M, Rossi F (2020). Modulation of circRNA Metabolism by m(6)A Modification. Cell Rep.

[29] Yang Y, Fan X, Mao M, Song X, Wu P, Zhang Y (2017). Extensive translation of circular RNAs driven by N(6)-methyladenosine. Cell Res.

[30] Chen Y, Ling Z, Cai X, Xu Y, Lv Z, Man D (2022). Activation of YAP1 by N6-Methyladenosine-Modified circCPSF6 Drives Malignancy in Hepatocellular Carcinoma. Cancer Res.

[31] Haskill S, Peace A, Morris J, Sporn SA, Anisowicz A, Lee SW (1990). Identification of three related human GRO genes encoding cytokine functions. Proc Natl Acad Sci USA.

[32] Sun J, Yuan J (2022). Chemokine (C-X-C motif) ligand 1/chemokine (C-X-C motif) receptor 2 autocrine loop contributes to cellular proliferation, migration and apoptosis in cervical cancer. Bioengineered.

[33] Kong W, Zhao G, Chen H, Wang W, Shang X, Sun Q (2021). Analysis of therapeutic targets and prognostic biomarkers of CXC chemokines in cervical cancer microenvironment. Cancer Cell Int.

[34] Man X, Yang X, Wei Z, Tan Y, Li W, Jin H (2022). High expression level of CXCL1/GROalpha is linked to advanced stage and worse survival in uterine cervical cancer and facilitates tumor cell malignant processes. BMC Cancer.

[35] Chinnaiyan P, Huang S, Vallabhaneni G, Armstrong E, Varambally S, Tomlins SA (2005). Mechanisms of enhanced radiation response following epidermal growth factor receptor signaling inhibition by erlotinib (Tarceva). Cancer Res.

[36] Tsai YC, Wang TY, Hsu CL, Lin WC, Chen JY, Li JH (2023). Selective inhibition of HDAC6 promotes bladder cancer radiosensitization and mitigates the radiation-induced CXCL1 signalling. Br J Cancer.

