# Supplementary material for: circRNF13, a novel N6-methyladenosine-modified circular RNA, enhances radioresistance in cervical cancer by increasing CXCL1 mRNA stability
Source: Cell Death Discov. 2023 Jul 20;9:253. doi: 10.1038/s41420-023-01557-0 (PMC10356927; doi:10.1038/s41420-023-01557-0)
Supplement: Supplementary file 1 — Supplementary table 1 [file 41420_2023_1557_MOESM1_ESM.doc]

**Supplementary table 1** **Sequences of primers used in the study**

| **Gene** | **Forward primer (5’-3’)** | **Reverse primer (5’-3’)** |
| --- | --- | --- |
| hsa_circ_0000021 | GTGGGATGACAGTGCGATTG | CAGCAGGCCCAGGTTCTC |
| hsa_circ_0001189 | TGAAGACACAGCTGGTTTCG | AGGCTGGCTTTTGATTCTGC |
| hsa_circ_0003239 | AAAGAAAAGTTGCCCGCTGT | AGGAGGTCGGAGGGGATAAT |
| hsa_circ_0008832 | CCACCAGCTCCAATCAGACT | AGTCCCCAGAAACAATCCCA |
| hsa_circ_0009061 | TCAAGAAGCAGCCTGTTTTCC | GCTTCTTTTCCTTCTCTGCTTTG |
| hsa_circ_0001346 | TTCATGATCACAAAATTTGTC | GTTTAAGAATGCAAAGAGCTGGA |
| hsa_circ_0044177 | AAGAGTCCAGACCATTGCGA | ATCAGGCCATCGTTCAGGG |
| CDK6 | GCTGACCAGCAGTACGAATG | GCACACATCAAACAACCTGACC |
| IL1B | ATGATGGCTTATTACAGTGGCAA | GTCGGAGATTCGTAGCTGGA |
| IGFBP3 | AGAGCACAGATACCCAGAACT | GGTGATTCAGTGTGTCTTCCATT |
| CXCL1 | CACAGTGTGTGGTCAACATTTC | AGTAAAGGTAGCCCTTGTTTCC |
