# Supplementary material for: circRNF13, a novel N6-methyladenosine-modified circular RNA, enhances radioresistance in cervical cancer by increasing CXCL1 mRNA stability
Source: Cell Death Discov. 2023 Jul 20;9:253. doi: 10.1038/s41420-023-01557-0 (PMC10356927; doi:10.1038/s41420-023-01557-0)

Figure 2F

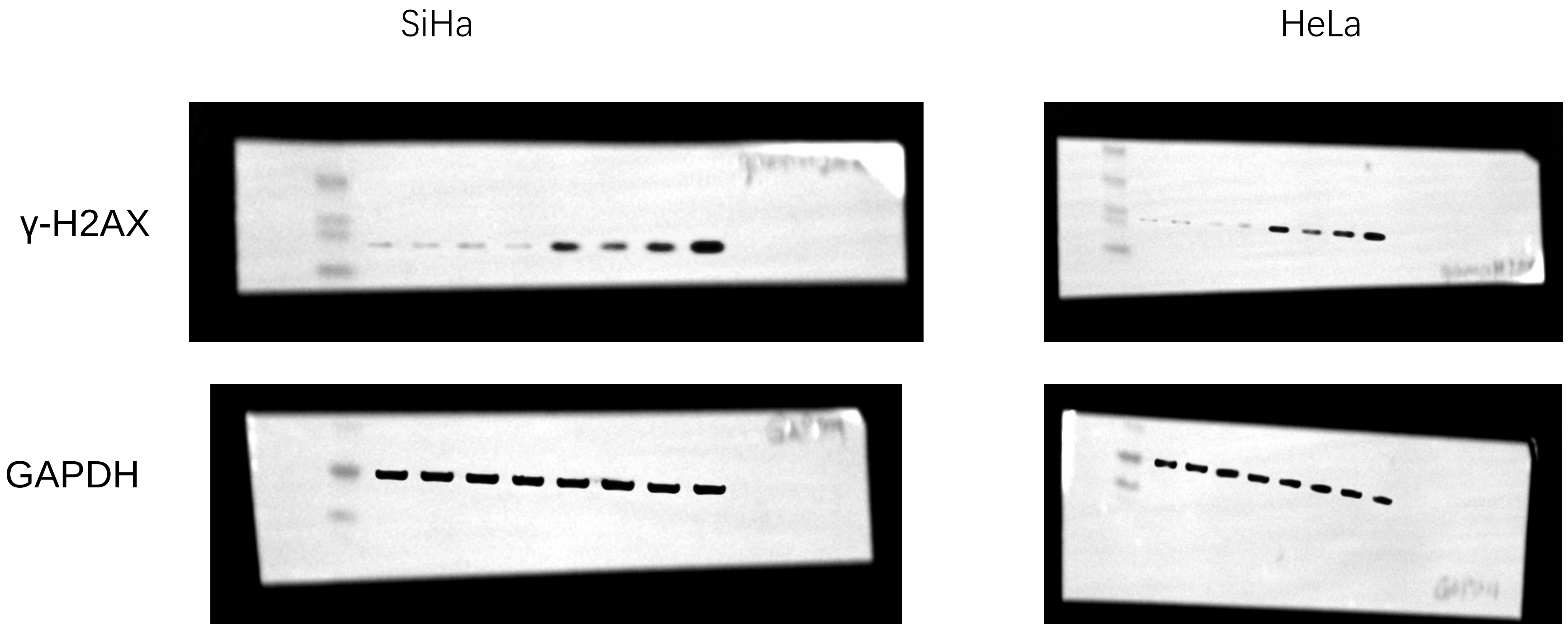

Figure 5D

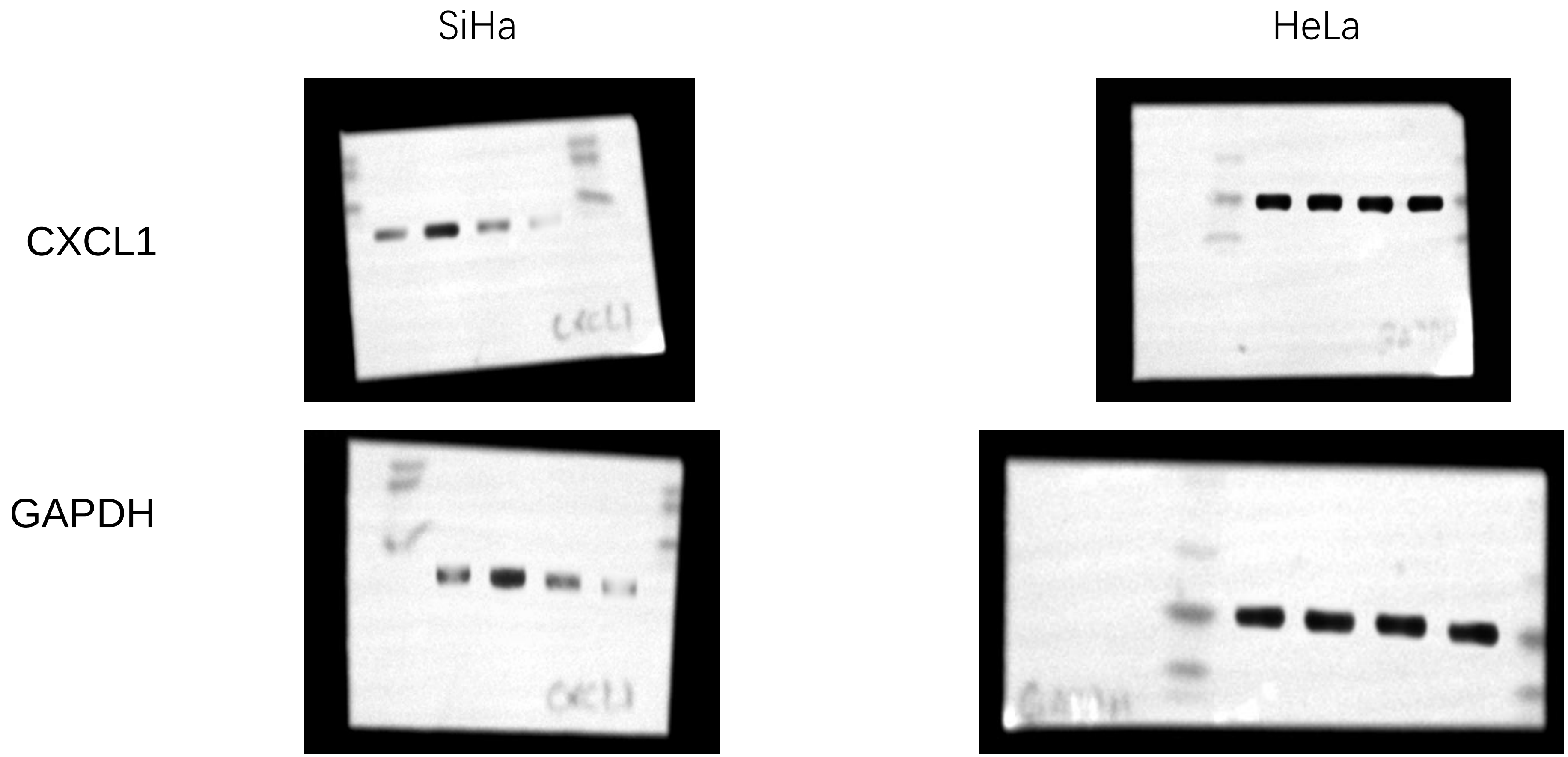

Figure 6B

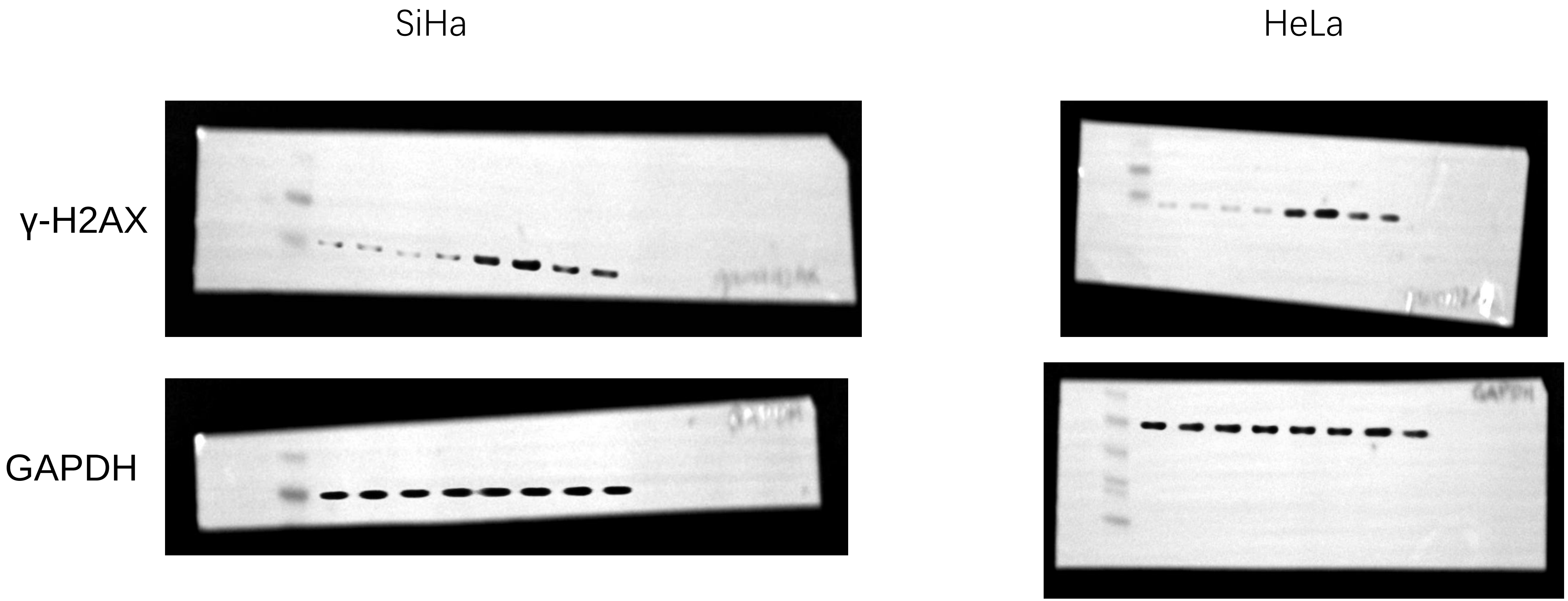

Supplement: Supplementary file 2 — Supplemental Material- Original Blots [file 41420_2023_1557_MOESM2_ESM.pdf]
